# Technic of Removing Completely Impacted Lower Third Molars, Requiring No Post-Operative Treatment

**Published:** 1918-03

**Authors:** James H. Howell

**Affiliations:** Detroit, Mich.


### TECHNIC OF REMOVING COMPLETELY IMPACTED LOWER THIRD MOLARS, REQUIRING NO POST-OPERATIVE TREATMENT

BY JAMES H. HOWELL, D.D.S., DETROIT, MICH.

1. At a previous sitting, the mouth was put into a clean aseptic condition by a prophylactic treatment.

2. Inject Novocain-Suprarenin, by the inferior mandibular technic and if the saliva is very profuse 1/160 of a grain of Atropin. This is given to dry up the saliva, by the time the Novocain-Suprarenin has taken effect the Atropin will also have done its duty.

3. Thoroughly wash the mouth with some antiseptic mouthwash and then with grain alcohol.

4. Place around the operative areas cotton rolls.

5. Paint the area with iodine.

6. Make a "J" shaped incision to the bone.

7. Lift the flap after carefully dissecting the periosteum away from the bone, under the flap, exposing the bone over the tooth.

8. Make two grooves, parallel, through the bone down to the tooth. One at the mesial side of the tooth and second at the distal side of the tooth. These grooves are made with a cross cut Fissure bur No. 560, and on the buccal side of the process.

9. The area of bone between the grooves can now be easily chiseled out, exposing the tooth.

10. The tooth can now be lifted out with an elevator.

11. The wound is carefully swabed out with sterile sponges, removing the bone grindings and blood.

12. Replace the flap of tissue with the periosteum on the under side and put one horsehair ligature at the curved part of the "J."

13. Carefully instruct the patient how to keep the parts clean with frequent washings of some antiseptic mouth wash.

14. Have the patient return in from 24 to 48 hours for you to remove the ligature.—*Journal N. D. A.*, for November.

